# Systematic Evaluation of the Application of Zebrafish in Toxicology (SEAZIT): Developing a Data Analysis Pipeline for the Assessment of Developmental Toxicity with an Interlaboratory Study

**DOI:** 10.3390/toxics11050407

**Published:** 2023-04-25

**Authors:** Jui-Hua Hsieh, Sue Nolte, Jon T. Hamm, Zicong Wang, Georgia K. Roberts, Charles P. Schmitt, Kristen R. Ryan

**Affiliations:** 1 Division of Translational Toxicology, National Institute of Environmental Health Sciences, Research Triangle Park, NC 27709, USA; zicong.wang@nih.gov (Z.W.); georgia.roberts@nih.gov (G.K.R.); 2 Office of Data Science, National Institute of Environmental Health Sciences, Research Triangle Park, NC 27709, USA; sue.nolte@nih.gov (S.N.); charles.schmitt@nih.gov (C.P.S.); 3 Inotiv, Morrisville, NC 27560, USA; jonathan.hamm@inotivco.com

**Keywords:** danio rerio, developmental toxicity testing, embryonic development, data analysis pipeline, phenotype ontology, developmental toxicants, interlaboratory comparisons

## Abstract

The embryonic zebrafish is a useful vertebrate model for assessing the effects of substances on growth and development. However, cross-laboratory developmental toxicity outcomes can vary and reported developmental defects in zebrafish may not be directly comparable between laboratories. To address these limitations for gaining broader adoption of the zebrafish model for toxicological screening, we established the Systematic Evaluation of the Application of Zebrafish in Toxicology (SEAZIT) program to investigate how experimental protocol differences can influence chemical-mediated effects on developmental toxicity (i.e., mortality and the incidence of altered phenotypes). As part of SEAZIT, three laboratories were provided a common and blinded dataset (42 substances) to evaluate substance-mediated effects on developmental toxicity in the embryonic zebrafish model. To facilitate cross-laboratory comparisons, all the raw experimental data were collected, stored in a relational database, and analyzed with a uniform data analysis pipeline. Due to variances in laboratory-specific terminology for altered phenotypes, we utilized ontology terms available from the Ontology Lookup Service (OLS) for Zebrafish Phenotype to enable additional cross-laboratory comparisons. In this manuscript, we utilized data from the first phase of screening (dose range finding, DRF) to highlight the methodology associated with the development of the database and data analysis pipeline, as well as zebrafish phenotype ontology mapping.

## 1. Introduction

The zebrafish embryo is a useful alternative research model for human disease. Approximately 70% of human genes have one or more orthologous zebrafish genes, and 82% of the genes that have morbidity descriptions listed in Online Mendelian Inheritance in Man (OMIM) have at least one zebrafish ortholog [1]. Additionally, the zebrafish embryo model is well-suited for chemical-induced toxicology assessments [2], including general toxicity [3,4], ecotoxicity [5,6,7], behavior toxicity [4,8,9], and developmental toxicity [10,11]. Compared to testing in mammals, zebrafish embryos have the benefits of a fast reproduction rate and a small, transparent body for the microscopic evaluation of altered phenotypes, making them amenable for higher throughput developmental toxicity screening [12].

In the past ten years, developmental toxicity screening using zebrafish embryos has been extensively investigated by OECD [13,14], by pharmaceutical companies [10,12,15,16], and by government-directed testing campaigns [17,18,19]. In the testing conducted by the pharmaceutical companies, the focus was to optimize the experimental conditions to achieve the best predictivity to mammalian data, using sets of 20 to 40 chemicals. Depending on the dataset composition, the predictivity, in comparison with the mammalian data, lies between 60% and 80%. In contrast, government-directed screenings such as the U.S. Environmental Protection Agency (US EPA) Toxicity Forecaster (ToxCast) [20] and the Developmental NeuroToxicity Data Integration and Visualization Enabling Resource (DNT-DIVER) hosted by the Division of Translational Toxicology (DTT, formerly Division of National Toxicology Program, DNTP) [19] (https://cebs.niehs.nih.gov/cebs/paper/13929, accessed on 10 April 2023) generally involved 100 to 1100 chemicals mostly with unknown developmental toxicity potential, and were intended as screens to gain toxicological information for prioritization.

Differences in experimental protocols have been shown to shift the potency of some chemicals, hence leading to scrutiny over the reliability of zebrafish embryonic assays to accurately assess developmental toxicity [21]. Similarly, other publications also highlight the need to further optimize experimental protocols and data analysis approaches [22,23]. In Beekhuijzen et al. and Song et al. [24,25], the authors intended to provide standardized experimental protocols with a standardized data analysis system to enhance data report harmonization. In Wilson et al. [26], eight substances were tested in four different experimental conditions and demonstrated, in some cases, the potency of substances could shift greatly. On the base of above efforts, the Systematic Evaluation of the Application of Zebrafish in Toxicology (SEAZIT) project program was developed in order to understand the influence of protocol differences on study outcome and to gain a broader adoption of the zebrafish developmental toxicity test (https://ntp.niehs.nih.gov/go/seazit, accessed on 10 April 2023).

One of SEAZIT’s primary objectives is to conduct an Interlaboratory Study across several organizations to investigate how experimental protocol parameters can influence the toxicity outcome (i.e., the concentration at which developmental toxicity is observed). As such, the Interlaboratory Study was specifically designed to understand how the dosing scenario (static vs. static renewal) and chorion status (chorion vs. de-chorion) influence chemical potency on embryo mortality and the development of altered phenotypes. For dosing scenarios, zebrafish were either dosed one time (dosing scenario = static or S) or were dosed every 24 h with media replacement (dosing scenario = static renewal or SR). Chorion status was also evaluated by either testing embryos with intact chorion (chorion status = chorion or C) or embryos subjected to a de-chorionation protocol (chorion status = de-chorion or DC). The two parameters, dosing scenario and chorion status, were previously identified by zebrafish experts to have a larger impact on the compound activity outcome, and thus were selected to be controlled in the screening [23].

The Interlaboratory Study has two phases: Dose Range Finding (DRF) and Definitive (Def). In DRF phase, each laboratory used the dosing scenario and chorion status in their respective in-house protocols. In the Def phase, each participating laboratory alternated the conditions of the two protocol parameters, thus four test conditions in total (i.e., S-C, SR-C, S-DC, SR-DC). The testing in Def phase is still ongoing. The goals of this manuscript are to describe the data management and data analysis pipeline development based on DRF data, as well as the preliminary findings based on DRF data. All the data were stored in a relational database using the open-source PostgreSQL data management system (https://www.postgresql.org/, accessed on 10 April 2023). The data were analyzed using the benchmark concentration (BMC) modeling approach, which we gained experience using in our recent publications on zebrafish developmental toxicity data from DNT-DIVER [21,27]. The BMC is similar to the concept of LOAEL (lowest-observed-adverse-effect level), but is not limited to tested concentrations, which is crucial when comparing results across laboratories.

In addition to the task of comparing the potency of identified developmental toxicants across laboratories, SEAZIT’s Interlaboratory study also sought to compare altered phenotypes induced by developmental toxicants across laboratories in terms of the sensitivity of substance-induced altered phenotypes. However, laboratories reported their findings using their own terminology. A recent publication of SEAZIT calls for greater uniformity in data reporting across laboratories [28]. Therefore, in this study, we mapped the altered phenotypes recorded using individual in-house laboratory terms to available zebrafish phenotype ontologies. This mapping allows the direct comparison of altered phenotypes between laboratories by translating findings into the same terminology.

In summary, the current study used the DRF data to develop a methodology associated with database development, the data analysis pipeline, and zebrafish phenotype ontology mapping. We will demonstrate the developed tools in the following sections.

## 2. Materials and Methods

### 2.1. Datasets

A dataset is defined as a screen conducted by one of the three laboratories (Lab-A, Lab-B, Lab-C) using one of the four controlled test conditions. The four controlled test conditions include Static Renewal-Chorion (SR-C), Static Renewal-Dechorion (SR-DC), Static-Chorion (S-C), and Static-Dechorion (S-DC). The screening includes two phases: Dose Range Finding (DRF) and Definitive (Def). In the DRF phase, each laboratory conducted the screens using the test condition that is commonly used in the respective laboratory; Lab-A used SR-C, Lab-B used S-DC, and Lab-C used S-C. Only the DRF data (i.e., three datasets; one from each of the three laboratories) are discussed within this manuscript. Data and analyses from the Def study will be forthcoming in a subsequent publication upon completion of the studies.

### 2.2. Screening Library

The screening library contains a total of 42 test substances, including one positive control (PC, 3,4-dichloroaniline), 3 blinded duplicates (aldicarb, bisphenol A, and valproic acid), and 35 screening substances. In total, there are 39 unique substances. Substances were dissolved in dimethyl sulfoxide (DMSO, 0.5–1%). The blinded substances were selected to represent a wide range of physicochemical properties: molecular weight (44.05 to 873.09), and partition coefficient (−3.01 to 6.7), and with consideration of available toxicological information, including the biological activity in rodent and zebrafish in vivo studies. More information regarding the substance selection is available in the companion manuscript [29]. To help group the compounds based on both use information and chemical structures, the level1 category and level2 category were created. The level1 category includes eight substance use groups (drug, flame retardant, fungicide, herbicide, industrial compound, insecticide, polycyclic aromatic hydrocarbon [PAH], preservative). The level2 category intends to add chemistry into the grouping; for example, the fungicide group (n = 6) can be split into element-organic fungicide (n = 3) and organic fungicide (n = 3). In the screening library, drug and insecticide are the two most representative groups, covering 28% (11/39) and 20.5% (8/39) of the compounds, respectively. The substance and its related category information is available in the Table S1 (sheet: substance). The screening library was prepared and maintained by DTT and provided to each laboratory.

### 2.3. Study Design for DRF

Substances were provided to each lab from the same provider (MRI Global, Kansas City, MO, USA) as concentrated stock to negate any variability rising from batch/lot variance. For exposure, the blinded substances, PC, and vehicle control (VC, 0.5% to 1% DMSO) were plated on a 96-well plate; each well contained one embryo. The representative plate map of each laboratory is provided in the Supplemental Figure S1. The maximum concentration for each substance was determined by DTT; the maximum concentration for most substances was 100 µM. Concentration spacing was different between laboratories. Lab-B used a smaller concentration spacing than Lab-A and Lab-C, resulting in more tested concentrations per substance: Lab-A and Lab-C tested 7 concentrations, with 11 embryos per concentration. Lab-B tested 11 concentrations with 7 embryos per concentration. All laboratories used 12 VC embryos per plate and 7 embryos (7 concentrations) for the PC per plate. Since only one embryo per concentration of PC was included on each plate, the PC data from plates run within a week were pooled for the benchmark concentration (BMC) analysis (see below). Each substance (a total of 41 substances, PC excluded) was screened in triplicate on separate dates (n = 3). The tabulated protocols of the three datasets in this study are provided in the Table S1 (sheet: protocol).

For each plate, embryos were treated with a test substance at 6 h-post-fertilization (hpf), and at both 24 hpf and 120 hpf, embryo mortality was recorded. If the embryo was viable at 120 hpf, the incidence of altered phenotypes was recorded under each laboratory’s individual protocols. The process of recording the incidence of substance-induced altered phenotypes was laboratory-specific, including naming conventions and varying altered phenotype sites of interest. Lab-A, B, and C recorded up to 21, 9, and 21 various altered phenotypes at 120 hpf in these three datasets, respectively, by either real-time visual inspection under a microscope (Lab-A and B) or based on inspecting captured 2-dimensional images and utilizing image analysis software (Lab-C). All recorded altered phenotypes in Lab-A and Lab-B, and twelve recorded altered phenotypes in Lab-C were binary phenotypes (presence or absence). The remaining recorded altered phenotypes in Lab-C were a binarized version of the quantitative physical traits (e.g., increased trunk length vs. VC). The names of the recorded altered phenotypes can be found in the Table S1 (sheet: ontology).

### 2.4. Zebrafish Phenotype Ontology Mapping

As mentioned above, each of the participating laboratories recorded a different number of altered phenotypes, which often have different names and can indicate different sites on the embryo. To compare the incidence of altered phenotypes across laboratories, we realized the importance of having a common language to translate the findings between laboratories. The Zebrafish Phenotype Ontology (https://www.ebi.ac.uk/ols/ontologies/zp, accessed on 10 April 2023) provides such a tool that formally defines phenotypes of the zebrafish model organism. After consulting with the laboratories, each of the altered phenotypes was mapped to one or multiple zebrafish phenotype ontology terms. For example, the ontology term/ID of Lab-A:Yolk_opacity was mapped to *yolk opaque, abnormal/ZP:0002198*. Since not all ontology terms were recorded by all the labs, to facilitate cross-lab comparison, we also grouped the ontology terms into descriptive developmental defect groupings. The groups of more well-defined developmental defects (granular) are sub-groups from more general developmental defects (general). For example, the *fin defects* group (in developmental defects (general)) has two sub-groups: *pectoral fin defects* and *caudal fin defects*. The complete mapping table is available in Table S1 (sheet: ontology).

### 2.5. Concentration-Response Data

For each of the recorded altered phenotypes, the incidence of either dead or malformed at each concentration of the substance on a plate was converted into a percent of response where the denominator was the total number of embryos, and the numerator was the sum of the outcome incidence. The resulting responses of a newly constructed endpoint combined both effects of a certain altered phenotype and mortality. For example, *Presence_of_head_Edema+Mort@120* endpoint represents the percent of response of the combined effect of mortality and head edema. Moreover, the *Mortality@120* (i.e., percent of mortality at 120 hpf) and *MalformedAny+Mort@120* endpoint (i.e., percent of affected embryo at 120 hpf) were calculated, where an affected embryo was an embryo that was either dead or malformed in any of the recordings. Only the altered phenotypes recorded as binary (presence or absence) were used in the calculation of *MalformedAny+Mort@120* endpoint. Adding a binary version of the quantitative physical traits to this endpoint in Lab-C resulted in inexplicable non-monotonic responses, and thus were excluded from the calculation of *MalformedAny+Mort@120* endpoint.

### 2.6. Benchmark Concentration (BMC) Modeling Data Analysis

The BMC approach was used to analyze the concentration-response data of endpoints. The BMC approach identifies the point-of-departure (POD) of the effect using a pre-defined threshold, benchmark response (BMR). The BMR in this analysis is interpreted as the lowest threshold that provides the best point estimation of the potency based on intrinsic data variance in an endpoint of a dataset. Specifically, a range of potential thresholds was explored, where each threshold represented the baseline noise level in the data. At each threshold, the pooled variance of potency of substances in the dataset was calculated after taking into account the noise in the data. The lowest threshold that provided a stabilized potency estimation was selected as the BMR. This was applied for each endpoint and applied to all laboratories. The whole process was described by [21,27] and was implemented in the function, ‘estimate_dataset_bmr’, in R package, *Rcurvep* (https://cran.r-project.org/web/packages/Rcurvep/index.html, accessed on 10 April 2023). The identified BMR for each endpoint is provided in the Table S1 (sheet: BMR). The derived BMR of each endpoint was applied to analyze the triplicate data of each substance separately to get three BMC values.

### 2.7. Altered Phenotype Specificity Analysis

The specificity score was calculated as the log10 difference between the BMC values of the mortality endpoint and a non-mortality endpoint (i.e., altered phenotype) at 120 hpf. A larger specificity score indicates a more specific effect compared with the potency for mortality. The specificity score threshold, which was used to separate a specific effect from a non-specific effect, was set as the 5th percentile of the distribution of the specificity score less than 0 in a dataset. In theory, the specificity score should be always larger than 0. However, in calculations, possibly due to the differences in dose spacing, BMR, and other intrinsic factors, there were cases where the specificity score was less than 0. Therefore, we propose that the distribution of the specificity score less than 0 can be used to estimate the noise in the BMC calculation for this data. The calculation of the specificity score was conducted at the independent plate level for each substance. Based on the potency and specificity score cutoff, three developmental toxicity classifications were assigned: specific (specificity score > cutoff), non-specific (specificity score ≤ cutoff), non-toxic (inactive in the endpoint). This information was then aggregated at the substance level (mostly with three separate plates except for the duplicates), and the majority class (>50%, usually 2 out of 3 plates) was decided. If there was no majority class (e.g., three different classes from three plates), the inconclusive class was assigned. The BMC and specificity score of the majority class were summarized using the mean. For inconclusive, the BMC and specificity score from the specific class were reported.

## 3. Results

In the following sections, we used the data of the DRF phase of the SEAZIT Interlaboratory Study to demonstrate how the data were stored in the database (Database section), the overall DRF data quality based on results in the vehicle control, positive control, and blinded duplicates (Quality check section), the endpoint variability between three laboratories in terms of the intrinsic response variation by BMR, and the BMC data of substances (Endpoint variability section). We also present the laboratory-specific altered phenotype to ontology mapping and developmental defects groupings (Zebrafish phenotype ontology mapping section). Finally, we demonstrate how we utilize mapped information to compare results across substances and laboratories based on developmental defect groupings (Altered phenotype specificity section).

### 3.1. Database

**Figure 1:**
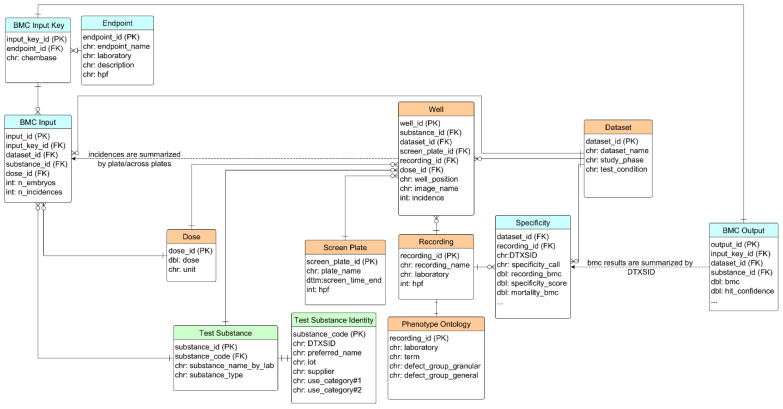
The entity relationship of tables in simplified SEAZIT database schema. The tables were put into three groups: substance (green), screening data (light orange), analyses (cyan). Only primary fields were shown in the tables. PK: primary key; FK: foreign key; chr: character; dbl: double; int: integer; dttm: date and time.

A relational database, called SEAZIT, was constructed using the PostgreSQL data management system. The tables in the database can be separated into three groups: *substance information*, *screening data*, and *analyses* (Figure 1). Starting with the test table comprising 42 substances, the identity information was stored in the table of test_substance_identity, including the fields of DTXSID (from US EPA Chemical Dashboard, https://comptox.epa.gov/dashboard/, accessed on 10 April 2023), lot, supplier, and use_category information. The *screening data* group includes six tables: well, dataset, recording, screen_plate, dose, and phenotype_ontology, where the well table is central in connection with others. The incidence data from the well data were summarized to the endpoint data for the table of BMC_input, either by plate (for blinded substances) or across plates (for PC). The table of BMC_input_key stores the unique combination of the endpoint and the aggregated chemical information. The input data were analyzed using the BMC modeling approach, and the generated BMC, BMR, and other relevant activity metrics were stored in the table of BMC_output. The specificity analysis was applied on the BMC_output data and the results were stored in the table of specificity and were linked back to the table of recording.

### 3.2. Quality Check

As part of the data analysis pipeline, data quality checks were conducted in three aspects: the reproducibility of the vehicle control (VC), positive control (PC), and blinded duplicates assessed by each laboratory. For the VC, the responses in three primary endpoints (*MalformedAny+Mort@120*, *Mortality@120*, *Mortality@24*) were compared. The VC responses in these three endpoints represent the baseline response.

**Figure 2:**
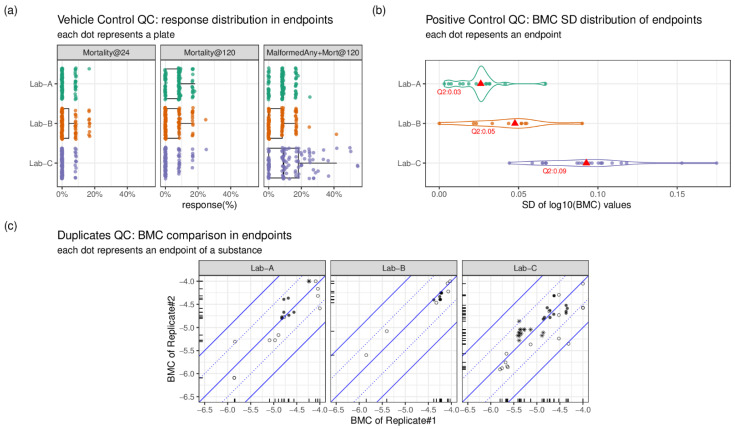
Quality check (QC) analysis for vehicle control (VC), positive control (PC), and duplicates (3 compounds, each of them has two substances) across three laboratories. (**a**) The response (%) distribution of three endpoints (*Mortality@24*, *Mortality@120*, *MalformedAny+Mort@120*) based on the response (%) from each plate of wells where embryos treated with only VC. (**b**) The distribution of standard deviation (SD) of the benchmark concentration (BMC) values of PC in all 120-hpf endpoints. Q2 represents the second quartile (median) of the distribution. (**c**) The BMC comparison of duplicates in all 120-hpf endpoints. The BMC is in log10(molar concentration) unit. The blue lines are diagonal lines with slope = 0, 1, −1 and intercept = 0. The blue dotted lines are diagonal lines with slope = 0.5, −0.5 and intercept = 0. The shape represents the three compounds: hollow circle as aldicarb; solid circle as bisphenol A; asterisk as valproic acid. Only BMC values different in replicate#1 and replicate#2 were kept in (**c**).

For endpoints of *Mortality@24* and *Mortality@120*, it was predetermined that the baseline response should be lower than 20%. All except two plates in the datasets of Lab-B and Lab-C met these criteria (Figure 2a). The exceptions (in mortality at 120 hpf) had only one additional dead embryo; thus, the plates were kept for analysis. Additionally, the response of the endpoint—percent of affected embryo at 120 hpf (*MalformedAny+Mort@120*) in the VC—was also checked to better understand the baseline level of altered phenotypes which occurred without chemical exposures. In Lab-A, only one plate had a response higher than 20% for this endpoint, while in Lab-B, only three plates had a response higher than 20% for this endpoint. In contrast, Lab-C had 26 plates with a response higher than 20% for this endpoint. The observation indicates that the baseline level of altered phenotypes is higher in Lab-C than the other two laboratories (Figure 2a).

For the PC, to understand the potency variation across the whole testing period of each laboratory, we calculated the standard deviation (SD) of BMC values of each endpoint. The collective SD values of the endpoints of each laboratory were plotted (Figure 2b). In total, 23, 11, and 23 endpoints’ SD values of PC BMC values were generated for Lab-A, Lab-B, and Lab-C, respectively. The median SD value of PC BMC values from the distributions for the Lab-A and Lab-B was 0.03 (1.07-fold) and 0.05 (1.12-fold), respectively; slightly smaller than the one in the Lab-C (0.09, 1.23-fold). Overall, the potency of the PC was reproducible across the testing period in all three laboratories, and the potency shift (as evaluated by the SD) was generally within 1.5-fold.

The inclusion of three blinded duplicates provides an additional quality check. It is expected that the BMC values from the duplicates (Replicate#1 and Replicate#2) can be similar. For each endpoint, the median BMC value from the triplicate testing (which is analyzed separately see Methods) was plotted, where the BMC value of Replicate#1 and the BMC value of Replicate #2 are the x and y axes, respectively (Figure 2c). If the point lands on the diagonal line through the origin, it signifies perfect BMC concordance. Additionally, two diagonal lines indicating 0.5 log10 potency (~3.2-fold) shift and 1 log10 (10-fold) potency shift were added to Figure 2c. All the points from Lab-B landed within the 0.5 log10 potency shift lines. By contrast, 2 points from Lab-A and 5 points from Lab-C exceeded the 0.5 log10 potency shift lines. The two points from Lab-A were related to the *Smaller_abnormal_eye_shape+Mort@120* and *Smaller_abnormal_head_shape+Mort@120* endpoints from aldicarb. The five endpoints from Lab-C were related to *Yolk_sac__Edema+Mort@120 (aldicarb)*, *Percardial_tissue__Edema+Mort@120 (aldicarb)*, *Pigment__abnormal+Mort@120 (aldicarb)*, *Pigment__decreased+Mort@120 (aldicarb)*, and *necrosis+Mort@120 (valproic acid)*. Overall, the BMC results of the blinded duplicates were reproducible, where the BMC difference between two replicates is generally within 0.5 log10 unit (~3.2 fold).

### 3.3. Endpoint Variability

Once all the endpoint data were collected in the database for each endpoint of datasets from three different laboratories, a benchmark response (BMR) value was derived (see Methods). For each dataset, 23, 11, and 23 endpoints (120-hpf) were generated for Lab-A, Lab-B, and Lab-C respectively. The derived BMR value for each endpoint is available in the Table S1 (sheet: BMR). To understand the diversity of the endpoints in each laboratory in terms of their intrinsic response variation, the distribution of the BMR values of the endpoints per dataset (laboratory) was plotted in the Supplemental Figure S2. The average BMR across all endpoints and chemicals and standard deviation (SD) for each dataset (laboratory) are 27% (2.5%), 37.3% (2.6%), and 32% (5.8%), for Lab-A, Lab-B, and Lab-C, respectively. Overall, endpoints in Lab-A have smaller BMRs than the other two laboratories, implying smaller intrinsic response variation in the endpoints of Lab-A, and that the endpoints are more homogeneous. Endpoints in Lab-C were observed to have more diverse BMR values, indicating the types of recordings are more heterogeneous.

**Figure 3:**
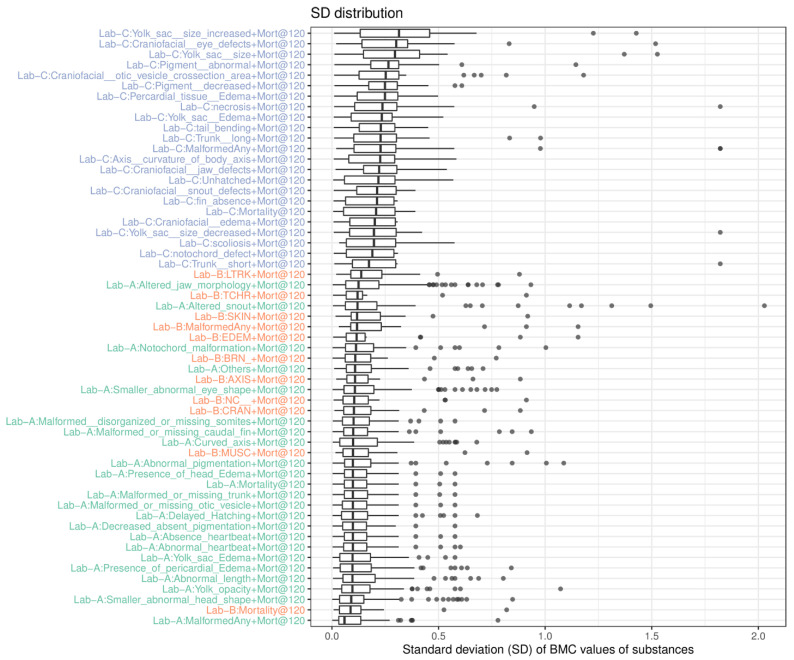
The distribution of the standard deviation (SD) values (dot) of BMC values of substances of endpoints in three laboratories (Lab-A: green; Lab-B: orange; Lab-C: blue). The endpoints were sorted based on the median value of the SD distribution.

The second analysis we conducted was to understand endpoint variability across three laboratories based on the associated benchmark concentration (BMC) data of substances, which were derived using the respective BMR values. Since each substance was tested in triplicate and was analyzed separately, the SD of the BMC values of a substance was calculated to represent the BMC variation. If the response was inactive, the highest tested concentration was used in the SD calculation. The collective SD values of each endpoint (only value > 0, i.e., active in at least one run) were plotted (Figure 3). We observed that the BMC data of triplicates from endpoints in Lab-A are least variable, followed by Lab-B and Lab-C. The analysis also indicates that the BMC data of the endpoints in Lab-A are more reliable than Lab-B, and Lab-C.

### 3.4. Zebrafish Phenotype Ontology Mapping

To facilitate the results comparison between three laboratories, the recorded altered phenotypes of each laboratory were mapped to one or multiple ontology terms. The relationship between the recorded altered phenotype and the mapped ontology term is 1 to 1 in all the mappings in Lab-A and Lab-C. For Lab-B, one recorded altered phenotype can have multiple mapped ontology terms. The average number of ontology terms for each recorded altered phenotype is 1.72 (19 ontology terms/11 recorded altered phenotypes), indicating less specificity in the recorded altered phenotypes. For example, *EDEM* (a recorded altered phenotype from Lab-B) was mapped to 2 ontology terms, including *heart edematous, abnormal* and *yolk edematous, abnormal*. Discussions with Lab-B indicated that combining altered phenotype types was conducted for the purposes of screening for developmental toxicity.

**Figure 4:**
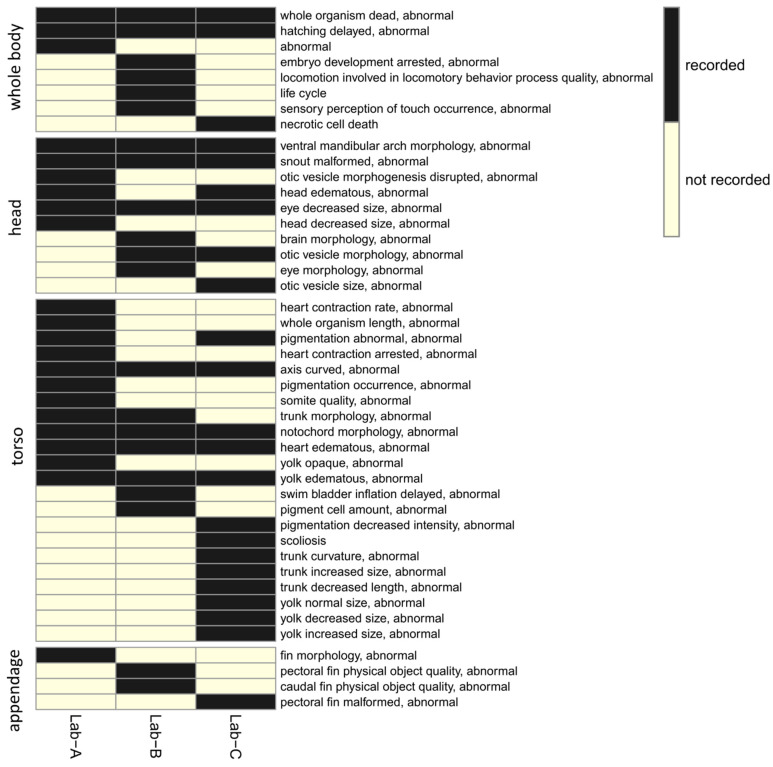
The comparison of ontology terms that were recorded or non-recorded across three laboratories. The terms were organized (and were separated into groups on the heatmap) based on location: whole body, head, torso, appendage.

**Figure 5:**
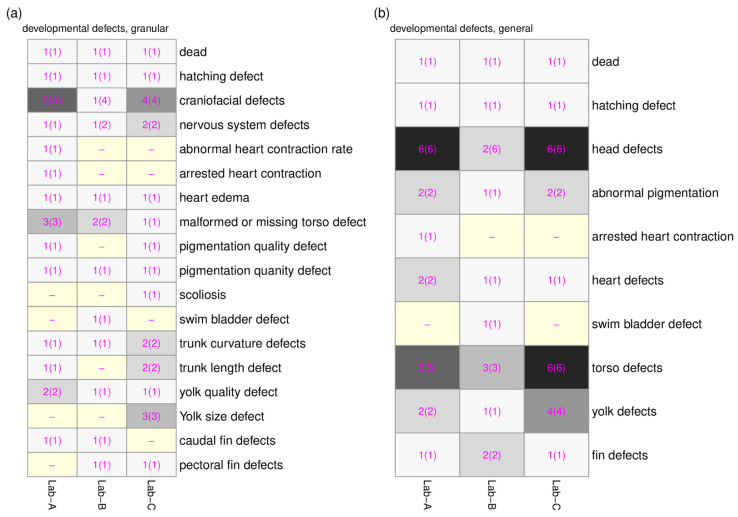
The comparison of the developmental defect groupings with number of associated recorded altered phenotypes and ontology terms across three laboratories. (**a**) developmental defects (granular). (**b**) developmental defects (general). The number in the cell represents the number of associated recorded altered phenotypes, and the number of associated ontology terms are in the parenthesis. The gray shading emphasized the number of recorded altered phenotypes (i.e., the darker gray color represents a higher number). The light-yellow color of the cell highlights the laboratory that did not have the associated developmental defect group recorded.

For all 44 ontology terms, only 8 ontology terms (whole organism dead, abnormal; hatching delayed, abnormal; ventral mandibular arch morphology, abnormal; snout malformed, abnormal; eye decreased size, abnormal; axis curved, abnormal; notochord morphology, abnormal; heart edematous, abnormal; yolk edematous, abnormal, in Figure 4) were recorded by all three laboratories, indicating that the recorded altered phenotypes between laboratories had limited overlap (Figure 4). The lack of overlap prompted us to create higher level ontology groups (developmental defects (granular) and developmental defects (general)). In total, 18 groups and 10 groups were created for developmental defects (granular) and developmental defects (general), respectively. The overlaps of the ontology groups between three laboratories were plotted using the heatmaps in Figure 5a,b. With the ontology groups, 9 out of 18 groups (developmental defects (granular)) and 8 out of 10 groups (developmental defects (general)) have a complete overlap across three laboratories. These additional groupings allow for comparative analysis of chemical-induced altered phenotypes across all three laboratories.

**Figure 6:**
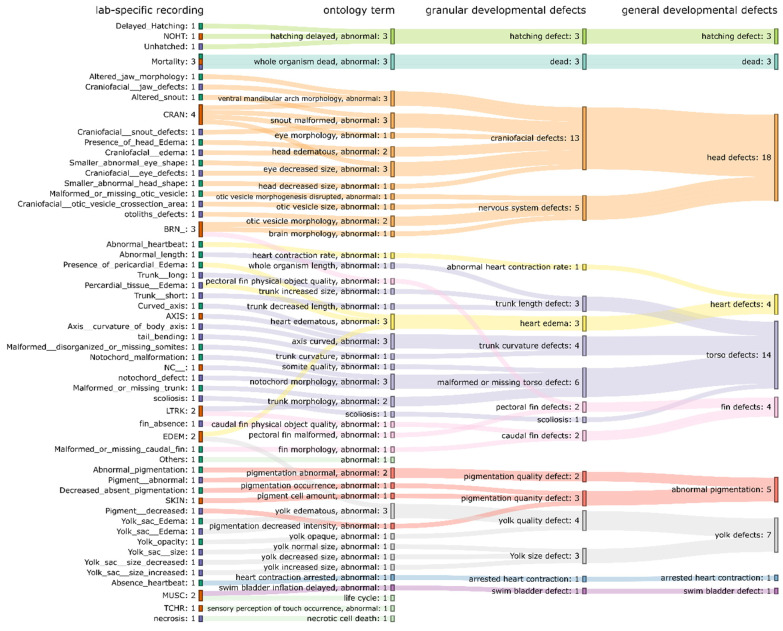
The hierarchical relationship of zebrafish altered phenotype ontology. The nodes from left to right are recorded altered phenotypes, ontology terms, developmental defects (granular), developmental defects (general). The color of recording nodes indicates the laboratory: green for Lab-A, orange for Lab-B, purple for Lab-C. The colors of the remaining nodes and flows indicate types from the highest hierarchy (developmental defects (general)). The number near each node summarizes the number of the incoming/outcoming flows. The number near the node of recorded altered phenotype indicates the number of the ontology terms associated with this recorded altered phenotype. The number near the node of ontology term indicates the number of laboratories having a recorded altered phenotype associated with this ontology term (maximum = 3 laboratories). The number near the node of either developmental defects (general) or developmental defects (granular) indicates the number of recorded altered phenotypes from all the laboratories associated with this group. Three ontology terms (light-green color, in the bottom of the plot) do not have the mapping of either developmental defects (general) nor developmental defects (granular) since they do not involve structural change.

To visualize the hierarchical relationship of components in zebrafish altered phenotype ontology (recordings from each laboratory → mapped ontology terms → developmental defects (granular) → developmental defects (general)), the Sankey diagram (Figure 6) was created. In Figure 6, we can see how each recorded altered phenotype is connected to the mapped ontology terms, then to ontology groups (developmental defects (granular) and developmental defects (general)). The *head defects* (in developmental defects (general)) contains the largest number of recorded phenotypes and includes a total of 18 recorded altered phenotypes from three laboratories, followed by torso defects (14 recorded altered phenotypes).

### 3.5. Altered Phenotype Specificity

The specificity score was calculated as the log10 difference between the BMC values of the mortality endpoint and a non-mortality endpoint (i.e., altered phenotype) at 120 hpf. A higher value of specificity score indicates the BMC of the non-mortality endpoint is lower than the BMC of the mortality endpoint. A threshold was applied to provide a binary specificity call, i.e., for a score larger than the threshold, as specific or otherwise non-specific. To obtain a specificity score threshold, the specificity score data of endpoints where the specificity score < 0 were collected per dataset (laboratory), and the 5th percentile of the distribution of the specificity score < 0 was set as the threshold. The distribution of specificity score < 0 was assumed to represent the noise distribution in the BMC calculation. Based on the distribution, the 5th percentile was observed to be 0.22, 0.03, and 0.14 for Lab-A, Lab-B, and Lab-C, respectively (Supplemental Figure S3), and was used as the specificity score threshold. The final specificity class of each altered phenotype was summarized at the compound level (see Methods). Overall, the percent of inconclusive calls was low and Lab-B had a higher percent of specific calls (Supplemental Figure S4).

**Figure 7:**
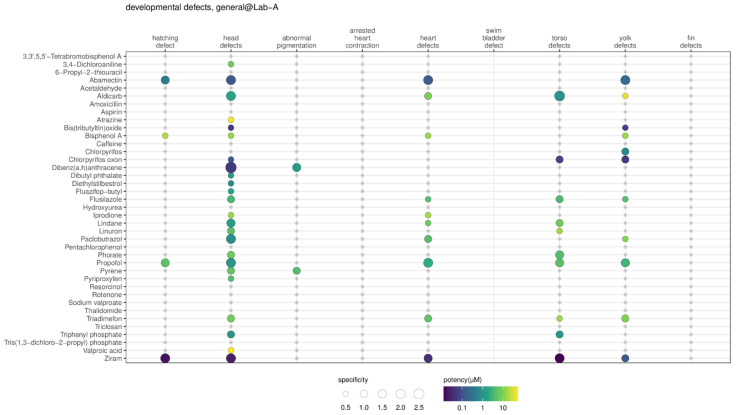
The summary of the substance-induced altered phenotypes for the dose ranging finding (DRF) study of Lab-A. Only results that are specific in the respective chemical-ontology group (in this example, the general developmental defects grouping) pair are shown as colored dots. The size of the dot represents the degree of specificity score, and the color represents the degree of potency. The gray asterisk (*) indicates the respective substance-ontology group was checked but was non-specific or non-toxic. More examples are available in the Supplemental Figure S6.

To compare the specificity results across laboratories, the recorded altered phenotypes were first mapped to the ontology terms, then the results of the ontology terms were collapsed into the groups of developmental defects. To select a representative result for each developmental defect group if multiple altered phenotypes were included, the results from the class of ‘specific’ were used first, followed by ‘non-specific’, ‘inconclusive’, and ‘non-toxic’. One example of data collapsing for head defects is shown in the Supplemental Figure S5 using a substance (abamectin) tested in Lab-A. To resolve ties (e.g., two ‘specific’ classes), the most potent BMC of the class was reported. The results are presented in Figure 7 and Figure S6. In Figure 7, the developmental defect group for head defects looked to be most sensitive in Lab-A, followed by yolk defects, heart defects, torso defects, hatching defects, and abnormal pigmentation. No substances (out of 39 substances) caused arrested heart contraction and fin defects in Lab-A DRF data. In Lab-C, the profile appeared to be similar to Lab-A, in that no substances caused fin defects. However, in Lab-B, the altered phenotype effects appeared to be more non-specific, and fin defects were also dominant (Supplemental Figure S6a,b).

## 4. Discussion

In this study, we constructed a relational database and evaluated the stored zebrafish embryo developmental toxicity data on 42 test substances from three laboratories which had distinct study design profiles. One aspect of the distinct study design profile is that laboratories used different experiment protocols in terms of the dosing scenario (S vs. SR) and chorion status (C vs. DC) in everyday laboratory practices: Lab-A uses SR-C, Lab-B uses S-DC, and Lab-C uses S-C. This phase of the study did not yield sufficient data to answer the question of which protocol parameter has the largest effect on substance-induced altered phenotypes; instead, we used the DRF data to design a database and to develop a data analysis pipeline. Based on the constructed database, we developed a data analysis pipeline to obtain the potency and specificity of substance-induced altered phenotypes. Even though the data were analyzed at the individual plate level, the results were summarized at the unique substance level, allowing users to quickly search for substance-induced specific toxicity. Additionally, to allow the comparison of specific altered phenotypes across laboratories, we mapped each lab-specific altered phenotype to an ontology term, then to more descriptive developmental defects groups. This is the first study to create tools for comparing zebrafish developmental toxicity results at the level of specific altered phenotypes rather than just the gross developmental toxicity across laboratories.

To investigate specific altered phenotypes, the specificity score threshold plays an important role in separating the specific developmental toxicity from non-specific developmental toxicity. The concept is similar to the ‘teratogenic index’ (TI, [12,15,16]. The TI is defined as LC_25_/NOAEL or LC_50_/EC_50_, where LC_25_/LC_50_ are the concentrations resulting in 25% or 50% lethality, respectively; and NOAEL/EC_50_ are the concentrations of the no-observed-adverse-effect level and 50% of the developmental toxicity effect, respectively. In this study, we opted to use the BMC approach to derive the point-of-departure of both lethality and a developmental toxicity effect, and to select the specificity cutoff based on dataset-specific statistics. The threshold in Lab-B was much smaller than Lab-A and Lab-C, possibly related to the smaller concentration spacing used in Lab-B. The average concentration spacing in Lab-B was less or equal to 0.2 vs. greater or equal to 0.5 in Lab-A and Lab-C. This exemplifies the impact of concentration spacing on the BMC approach used in this study, which will be important once we apply this approach to the definitive study (Def study).

The other aspect of the distinct laboratory study design profiles was that laboratories evaluate altered phenotypes differently. Lab-A and Lab-B only used visual inspection, but Lab-C used automated readouts from image analysis. Additionally, Lab-A and Lab-C focused more on distinguishing altered phenotypes in smaller regions, but Lab-B intended to capture overall effects in larger regions. This distinction was reflected in the number of mapped ontology terms to recorded altered phenotypes. For Lab-B, instead of a 1-to-1 relationship between the recorded altered phenotype and the ontology term as in Lab-A and Lab-C, on average, the relationship was 1:1.72. Additionally, when collapsing the recorded altered phenotype data into ontology groups, the results from separate ontology groups could come from the same recorded altered phenotype. The ontology mapping exercises possibly explained the non-specificity of the altered phenotype results of substances, as seen in Lab-B vs. other laboratories. Take the *fin defects* group for example; no substances caused this defect in Lab-A and Lab-C DRF data, but the defect was prominent in Lab-B. The observation may be explained by the mapping that the *fin defects* group linked to two recordings (BRN_ and LTRK), where BRN_ also linked to the most prominent defect group, *head defects*. So that if the substance caused a specific effect in the BRN_ recording, it would be considered to have a specific effect in the *fin defects* group. This is an inevitable outcome using the current recordings of Lab-B. Based on this observation, we might not be able to differentiate some regions of defects provided by Lab-B in the Def study. Examples were provided in Supplemental Figures S4 and S6, where Lab-B had a higher percentage of specific calls in altered phenotypes.

We believe that mapping ontology terms to individual altered phenotypes recorded in the laboratory can greatly improve the transferability of results between different laboratories. Therefore, we recommend that the zebrafish community adopts a similar practice for documenting their in-house altered phenotypes and provides this mapping in their publications. We also used the DRF data to understand the data variation in the datasets. The potency results from the endpoints in Lab-A appeared to be most consistent, followed by Lab-B, then Lab-C, suggesting that the potency from Lab-A is the most reliable. Having a lower variation of BMC values in triplicates will be advantageous in the Def phase to identify the protocol parameter contributing to the change of potency. All three datasets showed acceptable performance when doing the QC, based on VC, PC, and duplicates. However, in Lab-C, the baseline response level (VC response) was considerably higher than the other two laboratories, suggesting that it may be more difficult to identify a specific effect using the Lab-C DRF data. The same concern may be also applicable to the Lab-C Def dataset. We recommend the inclusion of multiple data quality checks, including the use of the blinded duplicates, as an important factor in the screening campaign.

In summary, by using the DRF data, we have completed the foundational work required to achieve the goals of SEAZIT: the development of a streamlined data analysis pipeline using the data stored in a relational database, and the harmonization of ontologies for altered phenotype endpoints provided by the laboratories. We are currently working on designing a web application to allow the user to interactively explore the data. The web application will also serve as the data portal for the Def data.

## Data Availability

The data are available in Chemical Effects in Biological Systems (CEBS) and can be downloaded through this link: https://doi.org/10.22427/NTP-DATA-002-00102-0001-000-6 (accessed on 10 April 2023).

## Supplementary Materials

The following supporting information can be downloaded at: https://www.mdpi.com/2305-6304/11/5/407/s1, Table S1 (sheet: protocol): tabulated protocol information; Table S1 (sheet: substance): substance identification data; Table S1 (sheet: ontology): phenotype ontology terms mapped to laboratory-specific recordings; Table S1 (sheet: BMR): BMR values of every endpoint. Figure S1. The representative 96-well plate map of the Lab-A (a), Lab-B (b), and Lab-C (c). The color represents the well type: vehicle control (gray); positive control (orange); a test substance (blue). The darker color represents a higher concentration. Figure S2. The distribution of benchmark threshold (BMR) values of 120-hpf endpoints of three laboratories (n = 23, 11, 23 for Lab-A, Lab-B, and Lab-C, respectively). The red dot is the mean value of the distribution (Lab-A: 26.96; Lab-B: 37.27; Lab-C: 31.96). Figure S3. The negative specificity score distribution. The red dot represents the 5th percentile and is used as the threshold. Figure S4. The percentage of developmental toxicity classifications of all phenotypes in a dataset. The # of phenotypes is 22, 10, 22 in Lab-A, Lab-B, and Lab-C, respectively and the # of chemicals is 39. Thus, the denominator is 858, 390, 858 for Lab-A, Lab-B, and Lab-C, respectively. Figure S5. The example of data collapsing starting from altered phenotypes to ontology terms, to granular developmental defect group, to general developmental defect group. Six altered phenotypes were associated with head defects. The concentration-response data of the six altered phenotypes after exposing to a test substance (abamectin) were presented, where green color represents the endpoint related to the altered phenotype and orange color represents the mortality endpoint. When the effect was specific, the yellow background was used. Figure S6. The summary of the chemical induced altered phenotypes for the dose ranging finding (DRF) study for three laboratories. Only results that are specific in the respective chemical-ontology group pair are shown as colored dots. The size of the dot represents the degree of specificity score, and the color represents the degree of potency. The gray asterisk indicates the respective chemical-ontology group is checked but the substance is non-specific or non-toxic in the assay. (a,b) Lab-B, and Lab-C results based on general developmental defects grouping. (c–e) Lab-A, Lab-B and Lab-C results based on granular developmental defects grouping. The plot for Lab-A results is presented in the Figure 7 in the main article.

## References

[1] Howe D.G., Bradford Y., Conlin T., Eagle A.E., Fashena D., Frazer K., Knight J., Mani P., Martin R., Moxon S.A.T. (2013). ZFIN, the Zebrafish Model Organism Database: Increased support for mutants and transgenics. Nucleic Acids Res..

[2] Polaka S., Koppisetti H., Pande S., Tekade M., Sharma M.C., Tekade R.K., Tekade R.K. (2022). Chapter 9-Zebrafish models for toxicological screening. Pharmacokinetics and Toxicokinetic Considerations.

[3] OECD (2013). Test No. 236: Fish Embryo Acute Toxicity (FET) Test, OECD Guidelines for the Testing of Chemicals, Section 2.

[4] Achenbach J.C., Leggiadro C., Sperker S.A., Woodland C., Ellis L.D. (2020). Comparison of the Zebrafish Embryo Toxicity Assay and the General and Behavioral Embryo Toxicity Assay as New Approach Methods for Chemical Screening. Toxics.

[5] Almond K.M., Trombetta L.D. (2016). The Effects of Copper Pyrithione, an Antifouling Agent, on Developing Zebrafish Embryos. Ecotoxicology.

[6] de Oliveira G.A.R., de Lapuente J., Teixidó E., Porredón C., Borràs M., de Oliveira D.P. (2016). Textile Dyes Induce Toxicity on Zebrafish Early Life Stages. Environmental Toxicology and Chemistry.

[7] Martins J., Oliva Teles L., Vasconcelos V. (2007). Assays with Daphnia Magna and Danio Rerio as Alert Systems in Aquatic Toxicology. Environ Int.

[8] Dasgupta S., Simonich M.T., Tanguay R.L. (2022). Zebrafish Behavioral Assays in Toxicology. Methods Mol Biol.

[9] Jarema K.A., Hunter D.L., Hill B.N., Olin J.K., Britton K.N., Waalkes M.R., Padilla S. (2022). Developmental Neurotoxicity and Behavioral Screening in Larval Zebrafish with a Comparison to Other Published Results. Toxics.

[10] Ball J.S., Stedman D.B., Hillegass J.M., Zhang C.X., Panzica-Kelly J., Coburn A., Enright B.P., Tornesi B., Amouzadeh H.R., Hetheridge M. (2014). Fishing for Teratogens: A Consortium Effort for a Harmonized Zebrafish Developmental Toxicology Assay. Toxicol Sci.

[11] Panzica-Kelly J.M., Zhang C.X., Augustine-Rauch K.A. (2015). Optimization and Performance Assessment of the Chorion-Off [Dechorinated] Zebrafish Developmental Toxicity Assay. Toxicol Sci.

[12] Brannen K.C., Panzica-Kelly J.M., Danberry T.L., Augustine-Rauch K.A. (2010). Development of a Zebrafish Embryo Teratogenicity Assay and Quantitative Prediction Model. Birth Defects Research Part B: Developmental and Reproductive Toxicology.

[13] Organization for Economic Cooperation and Development (2011). Series on Testing and Assessment: Ecotoxicity Testing, No. 157. Validation Report (Phase 1) for the Zebrafish Embryo Toxicity Test: Part I, Part II. https://www.oecd.org/chemicalsafety/testing/seriesontestingandassessmentecotoxicitytesting.htm.

[14] Organization for Economic Cooperation and Development (2012). Series on Testing and Assessment: Ecotoxicity Testing, No. 179. Validation Report (Phase 2) for the Zebrafish Embryo Tox-icity Test [Annexes]. https://www.oecd.org/chemicalsafety/testing/seriesontestingandassessmentecotoxicitytesting.htm.

[15] Gustafson A.-L., Stedman D.B., Ball J., Hillegass J.M., Flood A., Zhang C.X., Panzica-Kelly J., Cao J., Coburn A., Enright B.P. (2012). Inter-Laboratory Assessment of a Harmonized Zebrafish Developmental Toxicology Assay – Progress Report on Phase I. Reproductive Toxicology.

[16] Selderslaghs I.W.T., Blust R., Witters H.E. (2012). Feasibility Study of the Zebrafish Assay as an Alternative Method to Screen for Developmental Toxicity and Embryotoxicity Using a Training Set of 27 Compounds. Reproductive Toxicology.

[17] Padilla S., Corum D., Padnos B., Hunter D.L., Beam A., Houck K.A., Sipes N., Kleinstreuer N., Knudsen T., Dix D.J. (2012). Zebrafish Developmental Screening of the ToxCast^TM^ Phase I Chemical Library. Reproductive Toxicology.

[18] Truong L., Reif D.M., St Mary L., Geier M.C., Truong H.D., Tanguay R.L. (2014). Multidimensional In Vivo Hazard Assessment Using Zebrafish. Toxicol Sci.

[19] Behl M., Ryan K., Hsieh J.-H., Parham F., Shapiro A.J., Collins B.J., Sipes N.S., Birnbaum L.S., Bucher J.R., Foster P.M.D. (2019). Screening for Developmental Neurotoxicity at the National Toxicology Program: The Future Is Here. Toxicol Sci.

[20] Judson R.S., Houck K.A., Kavlock R.J., Knudsen T.B., Martin M.T., Mortensen H.M., Reif D.M., Rotroff D.M., Shah I., Richard A.M. (2010). In Vitro Screening of Environmental Chemicals for Targeted Testing Prioritization: The ToxCast Project. Environ Health Perspect.

[21] Hsieh J.-H., Behl M., Parham F., Ryan K. (2022). Exploring the Influence of Experimental Design on Toxicity Outcomes in Zebrafish Embryo Tests. Toxicol. Sci..

[22] Planchart A., Mattingly C.J., Allen D., Ceger P., Casey W., Hinton D., Kanungo J., Kullman S.W., Tal T., Bondesson M. (2016). Advancing Toxicology Research Using in Vivo High Throughput Toxicology with Small Fish Models. ALTEX.

[23] Hamm J.T., Ceger P., Allen D., Stout M., Maull E.A., Baker G., Zmarowski A., Padilla S., Perkins E., Planchart A. (2019). Characterizing Sources of Variability in Zebrafish Embryo Screening Protocols. ALTEX.

[24] Beekhuijzen M., de Koning C., Flores-Guillén M.-E., de Vries-Buitenweg S., Tobor-Kaplon M., van de Waart B., Emmen H. (2015). From Cutting Edge to Guideline: A First Step in Harmonization of the Zebrafish Embryotoxicity Test (ZET) by Describing the Most Optimal Test Conditions and Morphology Scoring System. Reprod Toxicol.

[25] Song Y.-S., Dai M.-Z., Zhu C.-X., Huang Y.-F., Liu J., Zhang C.-D., Xie F., Peng Y., Zhang Y., Li C.-Q. (2021). Validation, Optimization, and Application of the Zebrafish Developmental Toxicity Assay for Pharmaceuticals Under the ICH S5(R3) Guideline. Front. Cell Dev. Biol..

[26] Wilson L.B., Truong L., Simonich M.T., Tanguay R.L. (2020). Systematic Assessment of Exposure Variations on Observed Bioactivity in Zebrafish Chemical Screening. Toxics.

[27] Hsieh J.-H., Ryan K., Sedykh A., Lin J.-A., Shapiro A.J., Parham F., Behl M. (2019). Application of Benchmark Concentration (BMC) Analysis on Zebrafish Data: A New Perspective for Quantifying Toxicity in Alternative Animal Models. Toxicol Sci.

[28] Thessen A.E., Marvel S., Achenbach J.C., Fischer S., Haendel M.A., Hayward K., Klüver N., Könemann S., Legradi J., Lein P. (2022). Implementation of Zebrafish Ontologies for Toxicology Screening. Front. Toxicol..

[29] Hamm J.T., Hsieh J.-H., Roberts G., Collins B., Walker N.J., Ryan K. Systematic Evaluation of the Application of Zebrafish in Toxicology (SEAZIT): Inter-Laboratory Study Design.

