# Supplementary material for: Systematic Evaluation of the Application of Zebrafish in Toxicology (SEAZIT): Developing a Data Analysis Pipeline for the Assessment of Developmental Toxicity with an Interlaboratory Study
Source: Toxics. 2023 Apr 25;11(5):407. doi: 10.3390/toxics11050407 (PMC10222636; doi:10.3390/toxics11050407)
Supplement: Supplementary file 1 [file toxics-11-00407-s001.zip › toxics-2291086-supplementary_revised.pdf]

Systematic Evaluation of the Application of Zebrafish in Toxicology (SEAZIT): Developing a Data Analysis Pipeline for the Assessment of Developmental Toxicity with an Interlaboratory Study

Supplmental Figures

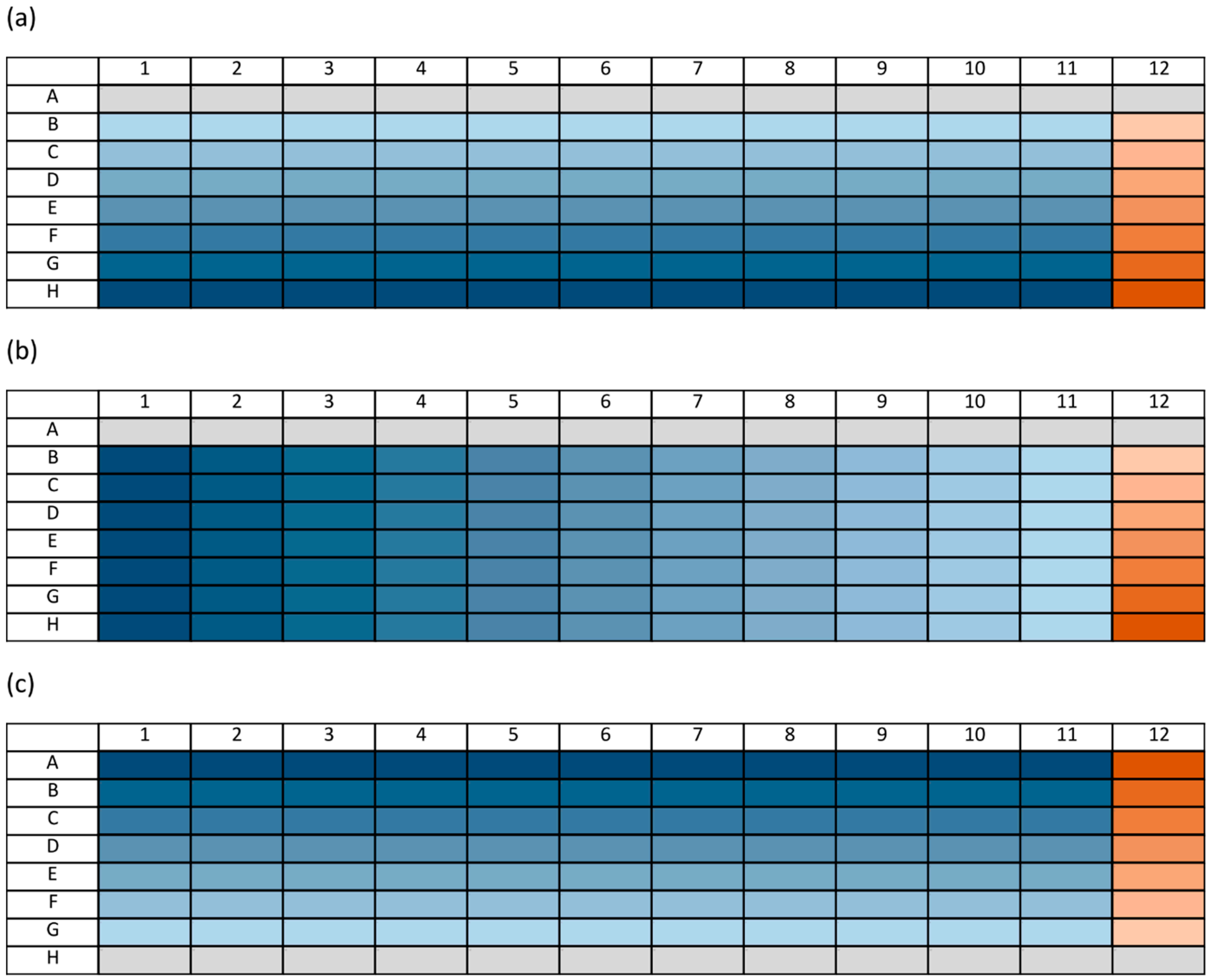

Supplemental Figure S1. The representative 96-well plate map of the Lab-A (a), Lab-B (b), and Lab-C (c). The color represents the well type: vehicle control (gray); positive control (orange); a test substance (blue). The darker color represents a higher concentration.

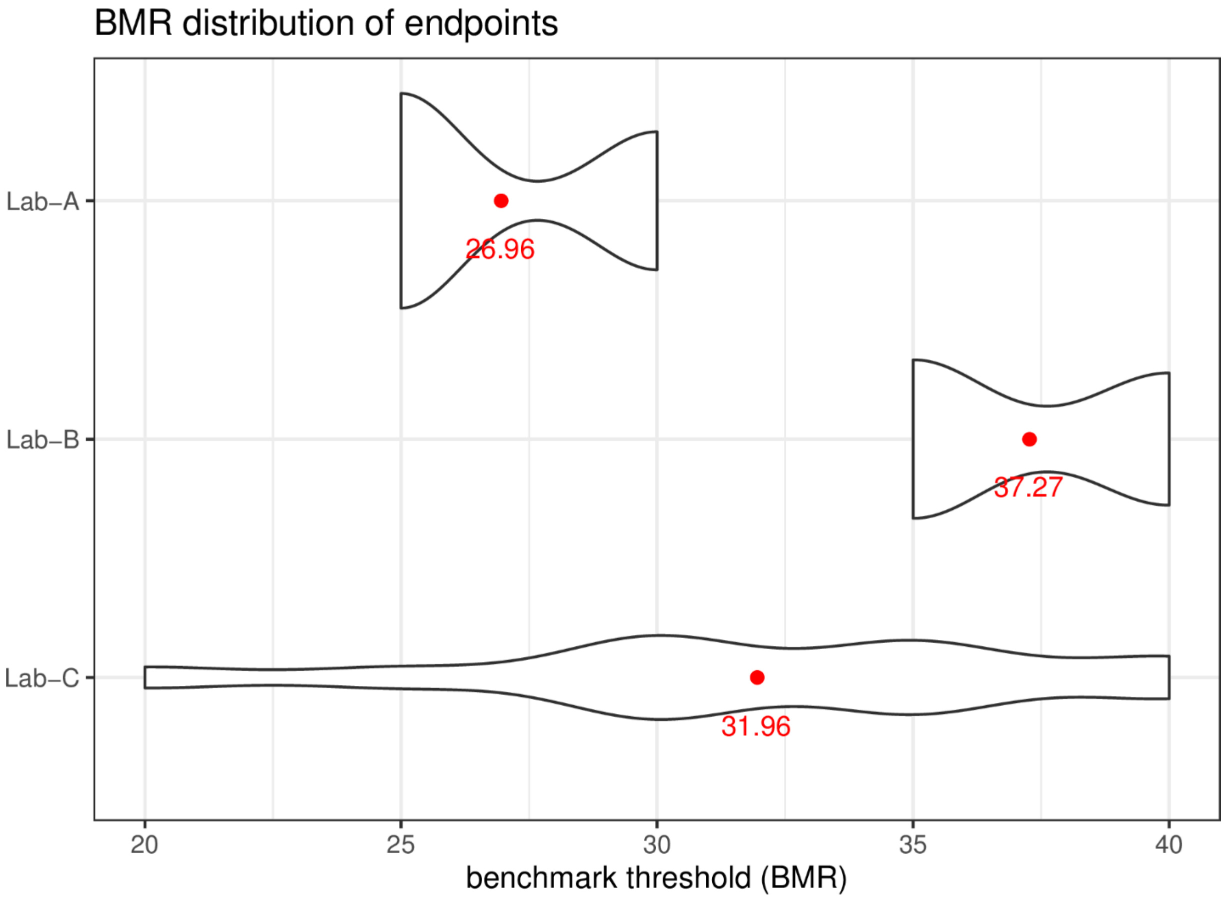

Supplemental Figure S2. The distribution of benchmark threshold (BMR) values of 120-hpf endpoints of three laboratories (n = 23, 11, 23 for Lab-A, Lab-B, and Lab-C, respectively). The red dot is the mean value of the distribution (Lab-A: 26.96; Lab-B: 37.27; Lab-C: 31.96)

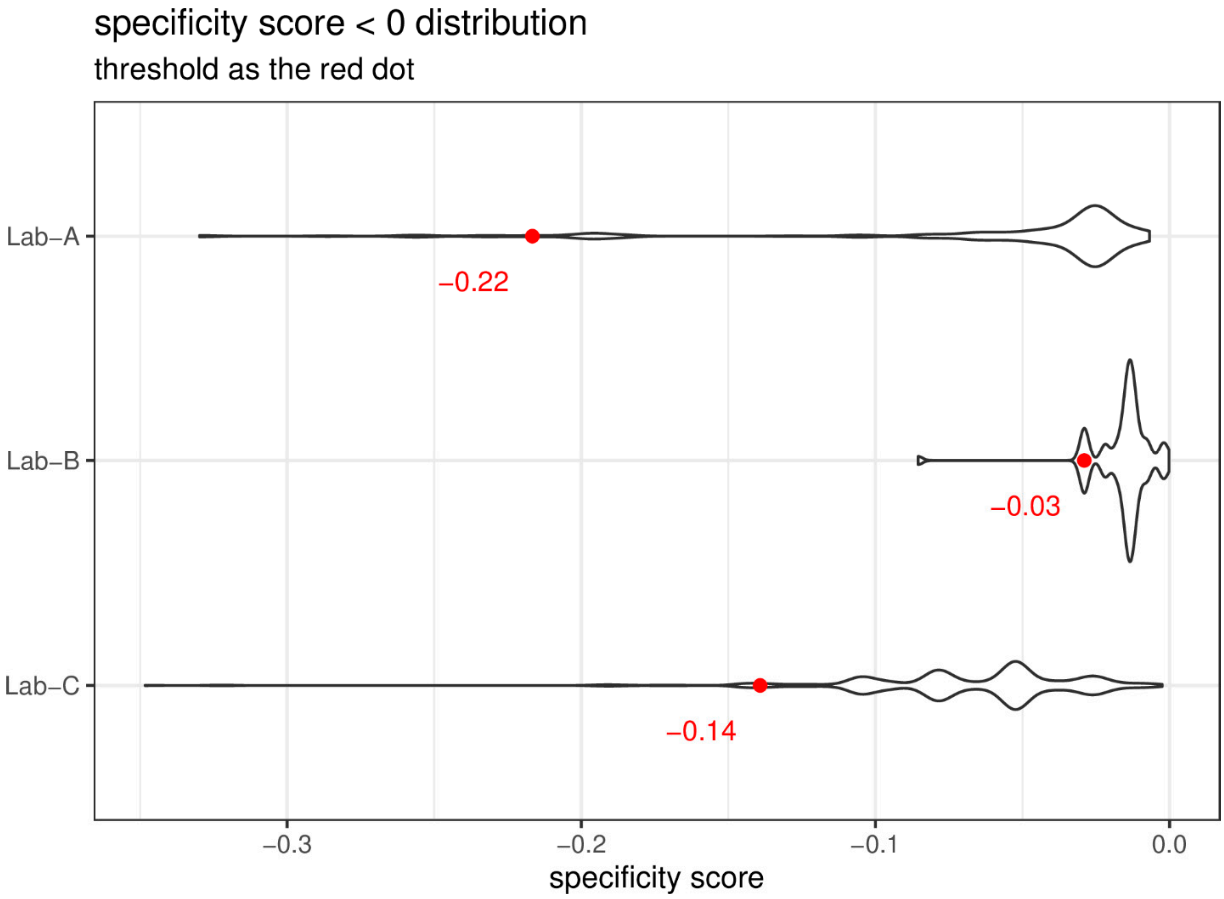

Supplemental Figure S3. The negative specificity score distribution. The red dot represents the 5th percentile and is used as the threshold.

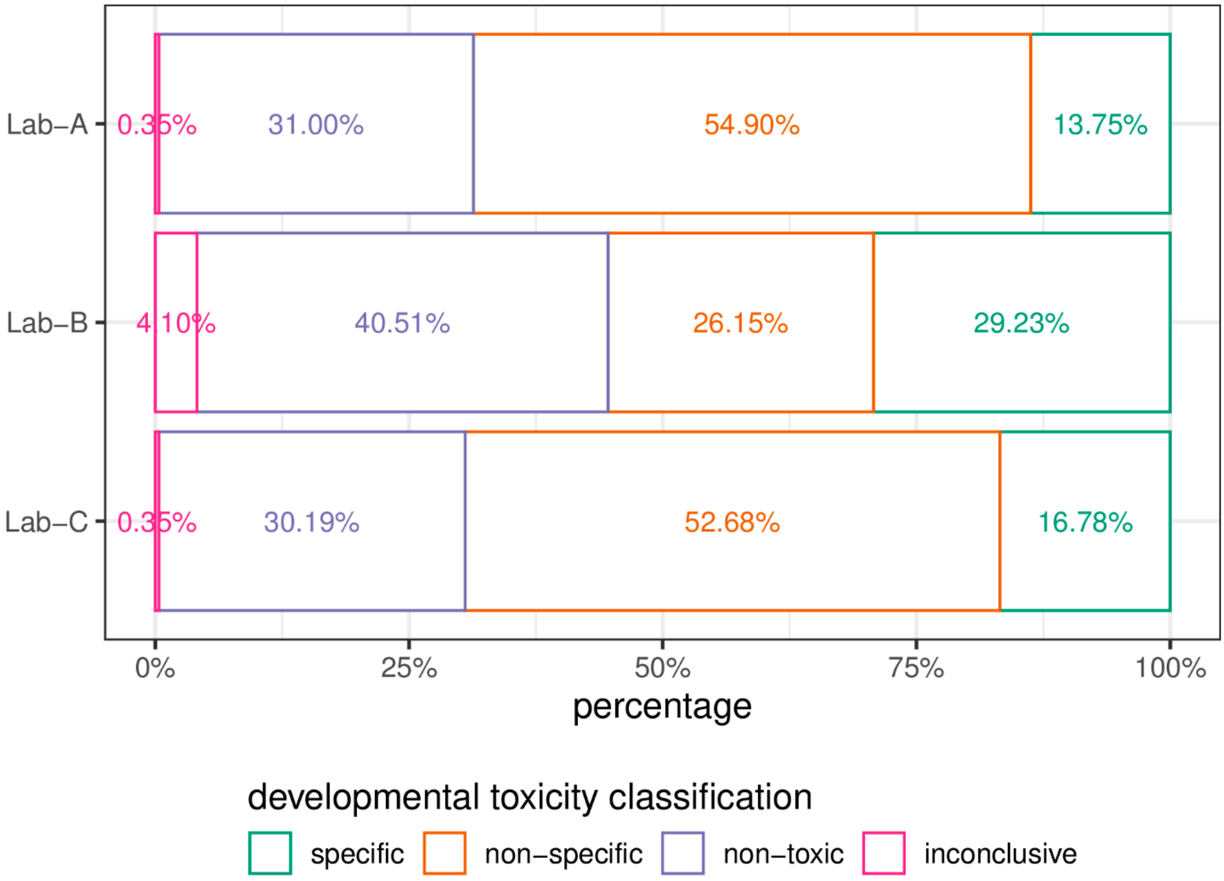

Supplemental Figure S4. The percentage of developmental toxicity classifications of all phenotypes in a dataset. The # of phenotypes is 22, 10, 22 in Lab-A, Lab-B, and Lab-C, respectively and the # of chemicals is 39. Thus, the denominator is 858, 390, 858 for Lab-A, Lab-B, and Lab-C, respectively.

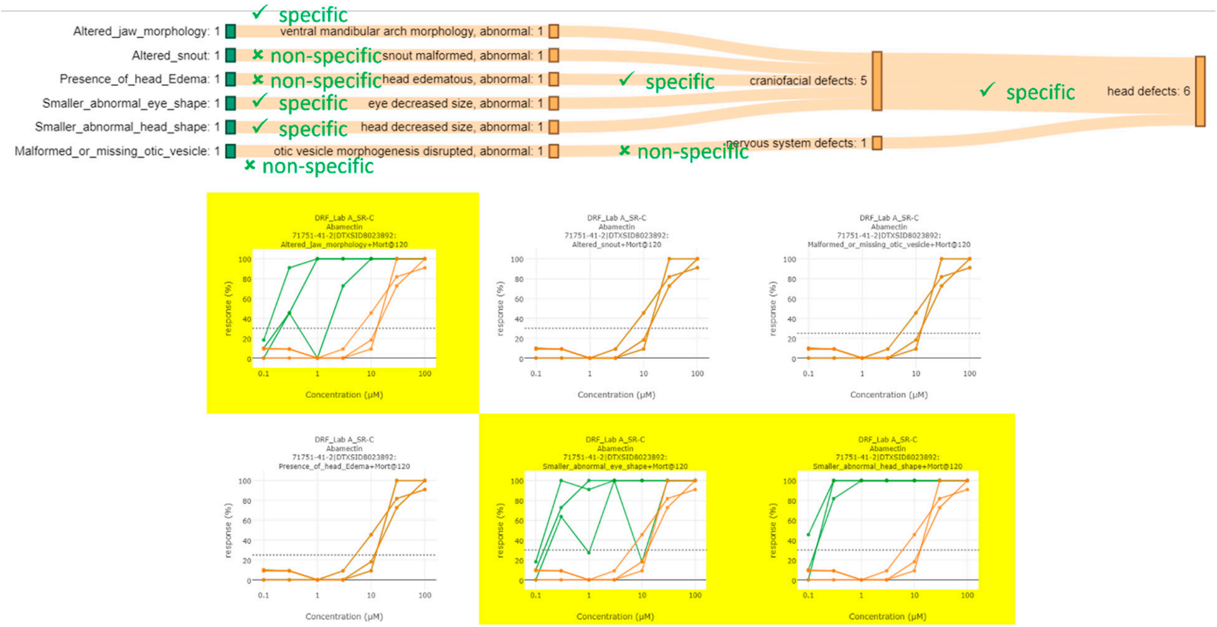

Supplemental Figure S5. The example of data collapsing starting from altered phenotypes to ontology terms, to granular developmental defect group, to general developmental defect group. Six altered phenotypes were associated with head defects. The concentration-response data of the six altered phenotypes after exposing to a test substance (abamectin) were presented, where green color represents the endpoint related to the altered phenotype and orange color represents the mortality endpoint. When the effect was specific, the yellow background was used.

(a)

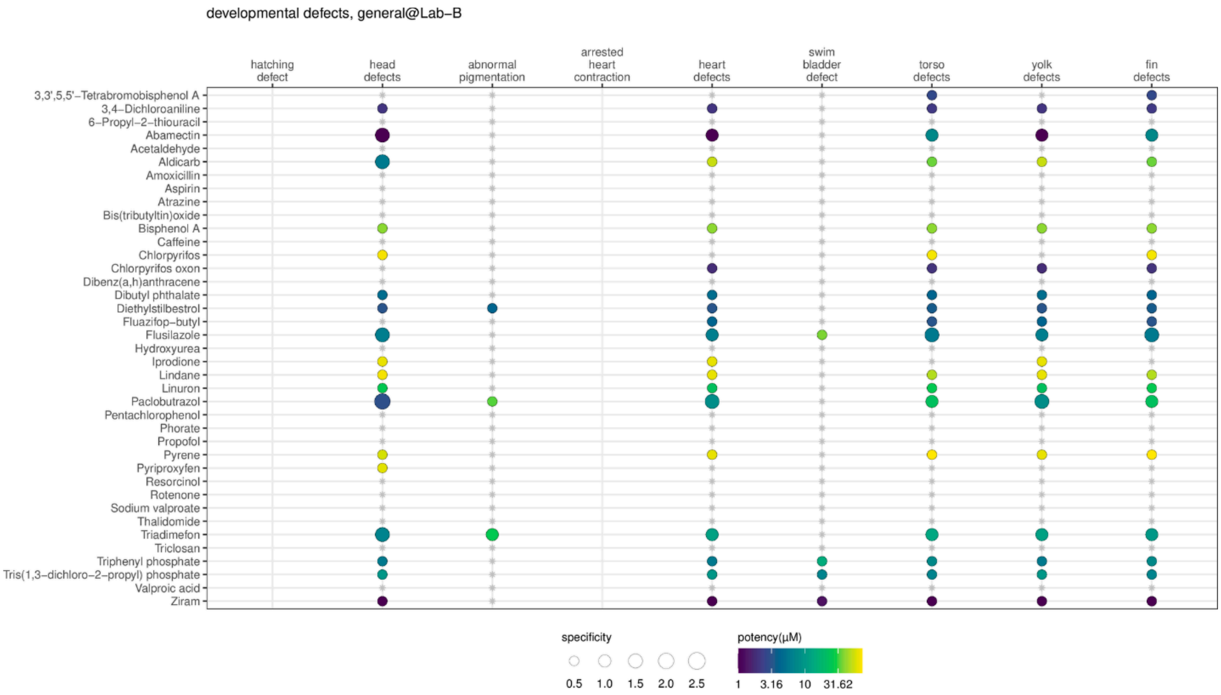
developmental defects, general@Lab-B
hatching defect
head defects
abnormal pigmentation
arrested heart contraction
heart defects
swim bladder defect
torso defects
yolk defects
fin defects
3,3',5,5'-Tetrabromobisphenol A
3,4-Dichloroaniline
6-Propyl-2-thiouracil
Abamectin
Acetaldehyde
Aldicarb
Amoxicillin
Aspirin
Atrazine
Bis(tributyltin)oxide
Bisphenol A
Caffeine
Chlorpyrifos
Chlorpyrifos oxon
Dibenz(a,h)anthracene
Dibutyl phthalate
Diethylstilbestrol
Fluazifop-butyl
Flusilazole
Hydroxyurea
Iprodione
Lindane
Linuron
Paclobutrazol
Pentachlorophenol
Phorate
Propofol
Pyrene
Pyriproxyfen
Resorcinol
Rotenone
Sodium valproate
Thalidomide
Triadimefon
Triclosan
Triphenyl phosphate
Tris(1,3-dichloro-2-propyl) phosphate
Valproic acid
Ziram
specificity
0.5 1.0 1.5 2.0 2.5
potency(µM)
1 3.16 10 31.62

(b)

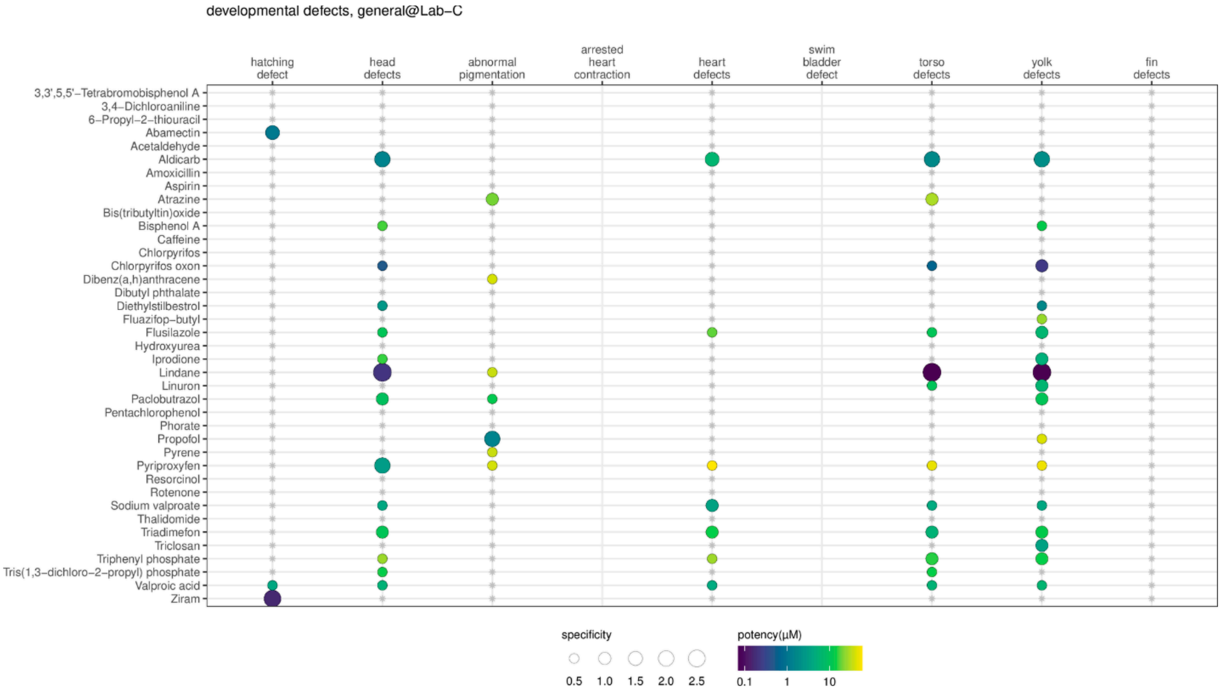
developmental defects, general@Lab-C
hatching defect
head defects
abnormal pigmentation
arrested heart contraction
heart defects
swim bladder defect
torso defects
yolk defects
fin defects
3,3',5,5'-Tetrabromobisphenol A
3,4-Dichloroaniline
6-Propyl-2-thiouracil
Abamectin
Acetaldehyde
Aldicarb
Amoxicillin
Aspirin
Atrazine
Bis(tributyltin)oxide
Bisphenol A
Caffeine
Chlorpyrifos
Chlorpyrifos oxon
Dibenz(a,h)anthracene
Dibutyl phthalate
Diethylstilbestrol
Fluazifop-butyl
Flusilazole
Hydroxyurea
Iprodione
Lindane
Linuron
Paclobutrazol
Pentachlorophenol
Phorate
Propofol
Pyrene
Pyriproxyfen
Resorcinol
Rotenone
Sodium valproate
Thalidomide
Triadimefon
Triclosan
Triphenyl phosphate
Tris(1,3-dichloro-2-propyl) phosphate
Valproic acid
Ziram
specificity
0.5 1.0 1.5 2.0 2.5
potency(µM)
0.1 1 10

(c)

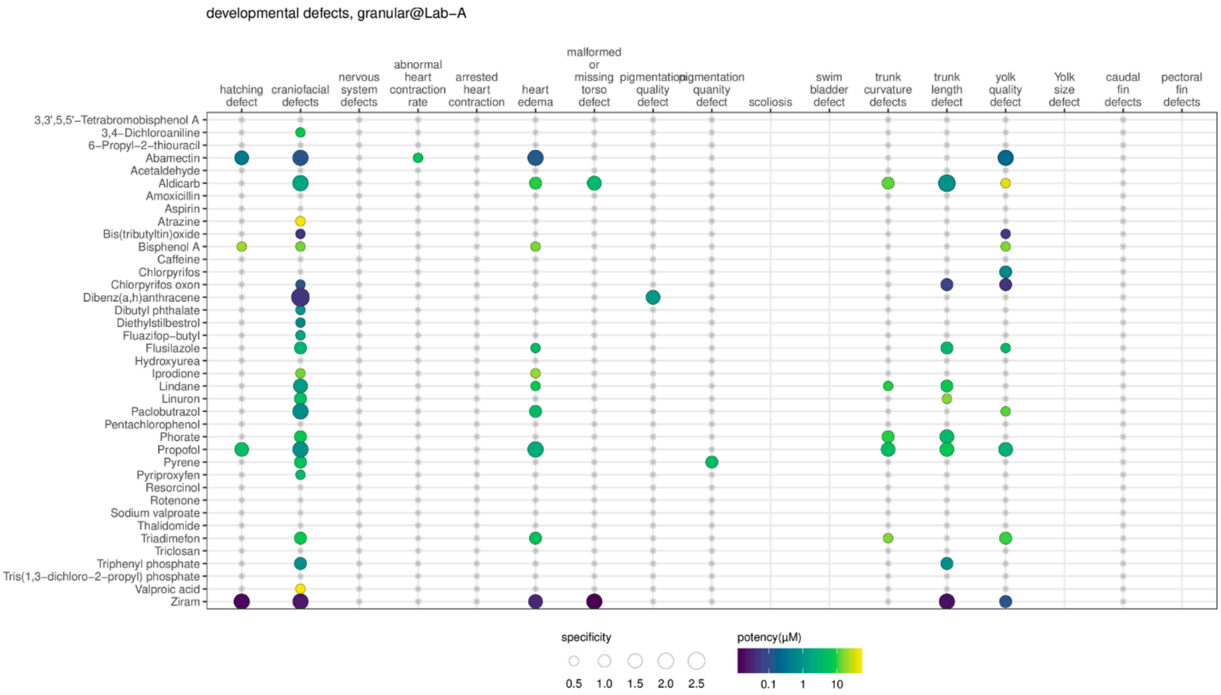
developmental defects, granular@Lab−A
hatching defect
craniofacial defects
nervous system defects
abnormal heart contraction rate
arrested heart contraction
heart edema
malformed or missing torso defect
pigmentation quality defect
pigmentation quanity defect
scoliosis
swim bladder defect
trunk curvature defects
trunk length defect
yolk quality defect
Yolk size defect
caudal fin defects
pectoral fin defects
3,3',5,5'−Tetrabromobisphenol A
3,4−Dichloroaniline
6−Propyl−2−thiouracil
Abamectin
Acetaldehyde
Aldicarb
Amoxicillin
Aspirin
Atrazine
Bis(tributyltin)oxide
Bisphenol A
Caffeine
Chlorpyrifos
Chlorpyrifos oxon
Dibenz(a,h)anthracene
Dibutyl phthalate
Diethylstilbestrol
Fluazifop−butyl
Flusilazole
Hydroxyurea
Iprodione
Lindane
Linuron
Paclobutrazol
Pentachlorophenol
Phorate
Propofol
Pyrene
Pyriproxyfen
Resorcinol
Rotenone
Sodium valproate
Thalidomide
Triadimefon
Triclosan
Triphenyl phosphate
Tris(1,3−dichloro−2−propyl) phosphate
Valproic acid
Ziram
specificity
0.5 1.0 1.5 2.0 2.5
potency(μM)
0.1 1 10

(d)

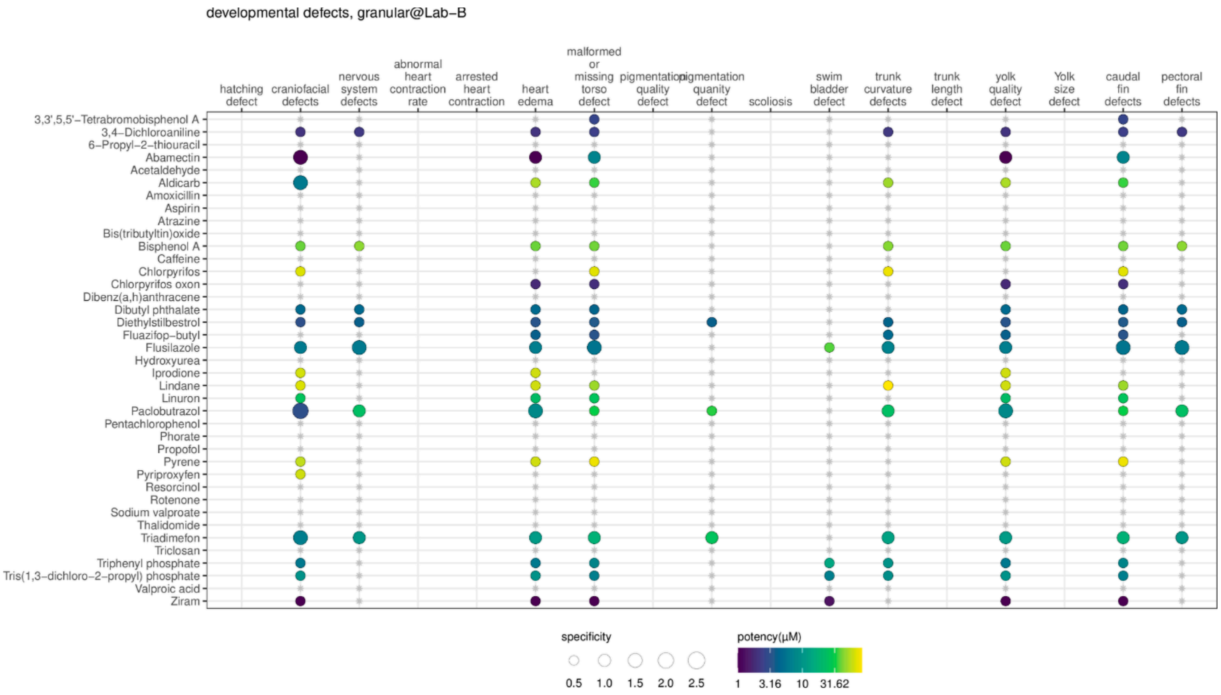
developmental defects, granular@Lab−B
hatching defect
craniofacial defects
nervous system defects
abnormal heart contraction rate
arrested heart contraction
heart edema
malformed or missing torso defect
pigmentation quality defect
pigmentation quanity defect
scoliosis
swim bladder defect
trunk curvature defects
trunk length defect
yolk quality defect
Yolk size defect
caudal fin defects
pectoral fin defects
3,3',5,5'−Tetrabromobisphenol A
3,4−Dichloroaniline
6−Propyl−2−thiouracil
Abamectin
Acetaldehyde
Aldicarb
Amoxicillin
Aspirin
Atrazine
Bis(tributyltin)oxide
Bisphenol A
Caffeine
Chlorpyrifos
Chlorpyrifos oxon
Dibenz(a,h)anthracene
Dibutyl phthalate
Diethylstilbestrol
Fluazifop−butyl
Flusilazole
Hydroxyurea
Iprodione
Lindane
Linuron
Paclobutrazol
Pentachlorophenol
Phorate
Propofol
Pyrene
Pyriproxyfen
Resorcinol
Rotenone
Sodium valproate
Thalidomide
Triadimefon
Triclosan
Triphenyl phosphate
Tris(1,3−dichloro−2−propyl) phosphate
Valproic acid
Ziram
specificity
0.5 1.0 1.5 2.0 2.5
potency(μM)
1 3.16 10 31.62

(e)

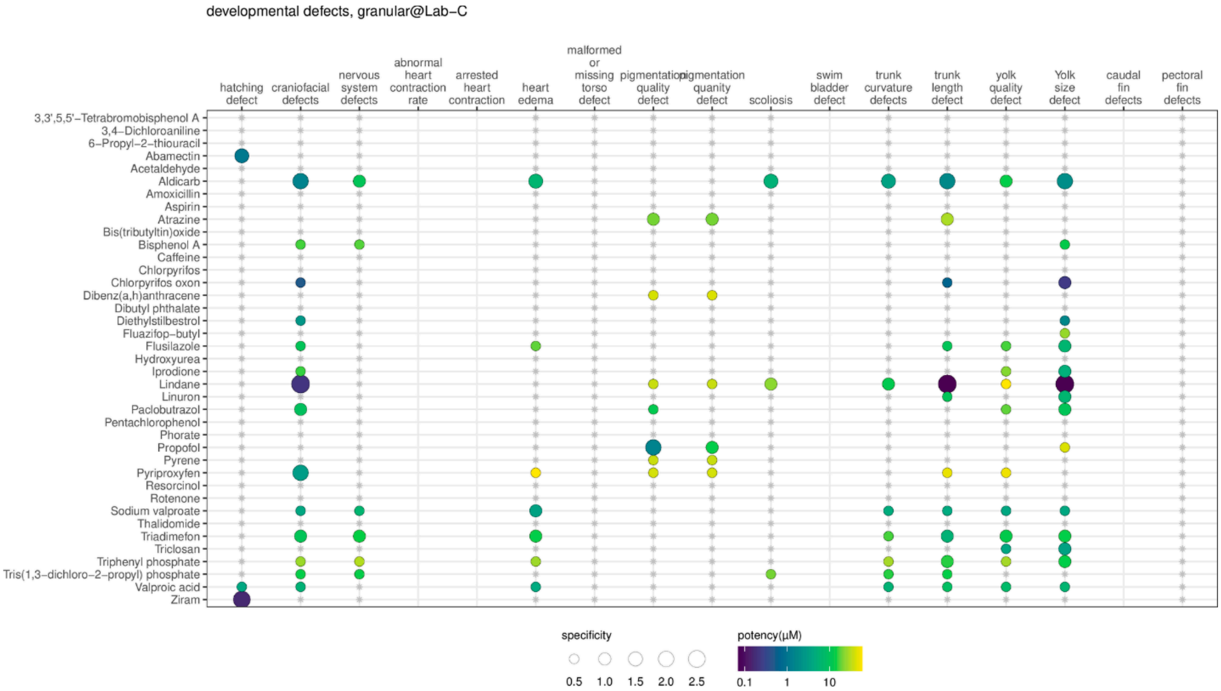

Supplemental Figure S6. The summary of the chemical induced altered phenotypes for the dose ranging finding (DRF) study for three laboratories. Only results that are specific in the respective chemical-ontology group pair are shown as colored dots. The size of the dot represents the degree of specificity score, and the color represents the degree of potency. The gray asterisk indicates the respective chemical-ontology group is checked but the substance is non-specific or non-toxic in the assay. a)-b) Lab-B, and Lab-C results based on general developmental defects grouping. c)-e) Lab-A, Lab-B and Lab-C results based on granular developmental defects grouping. The plot for Lab-A results is presented in the Figure 7 in the main article.
